# A simplified and reliable HPV testing of archival Papanicolaou-stained cervical smears: application to cervical smears from cancer patients starting with cytologically normal smears

**DOI:** 10.1054/bjoc.1999.1128

**Published:** 2000-03-21

**Authors:** M V Jacobs, D Zielinski, C J L M Meijer, R P Pol, F J Voorhorst, F A de Schipper, A P Runsink, P J F Snijders, J M M Walboomers

**Affiliations:** Department of Pathology, Section of Molecular Pathology, University Hospital Vrije Universiteit, De Boelelaan 1117, Amsterdam, 1081 HV, The Netherlands; Department of Epidemiology and Biostatistics, Fac. Geneeskunde Vrije Universiteit, Van der Boechorststraat 7, Amsterdam, 1081 BT, The Netherlands; Department of Obstetrics and Gynecology, Hospital Walcheren, Koudekerkseweg 88, Vlissingen, 4382 EE, The Netherlands; Department of Pathology, District Laboratory Zeeland, Molenwater 47, Middelburg, 4331 SC, The Netherlands

**Keywords:** HPV, PCR, archival Pap smears

## Abstract

The efficacy of four methods to recover DNA from Papanicolaou (Pap)-stained archival cervical smears for optimal detection of human papillomavirus (HPV) DNA by GP5+/bioGP6+ polymerase chain reaction (PCR) was investigated. Two of the methods were based on proteinase K treatment and two based on treatment with guanidinium thiocyanate (GTC). The quality of the DNA as measured by PCR assays amplifying different sizes of the β-globin gene appeared to be superior for the GTC-based assays. Using competitive β-globin PCR assays, one of the GTC-based, assays, provisionally named High Pure PCR Template Preparation (HPPTP) assay, yielded by far the highest quantity of amplifiable DNA. It allowed the recovery of 2.2 × 10^5^to 3 × 10^5^genome equivalents in smears containing 5 × 10^5^to 20 × 10^5^nucleated cells, indicating a mean efficiency of 26% (range of 15–44%). In contrast, the other methods revealed markedly lower efficiencies varying from 1% to 10%. The use of the HPPTP assay as a reliable processing procedure was validated by demonstrating a complete agreement in HPV detection and 93% agreement in HPV typing between 39 archival Pap-stained and paired fresh-frozen cervical smears. This method was applied to 40 archival smears from ten cervical cancer patients (selected from a group of 200 patients) which had a history of 3–6 smears with the first smear being Pap 1 or 2 taken at least 5 years before cancer was diagnosed. The average time period between the first Pap 1/2 smear that contained the same HPV type as in the corresponding carcinoma and diagnosis of cervical cancer was 12.0 ± 2.9 years. All subsequent smears were invariably positive for the same HPV type which was also found in the cervical cancer biopsy. In conclusion, the HPPTP assay provides a reliable and efficient means to extract DNA from Pap-stained archival cervical smears for the detection of HPV DNA by PCR and would be the method of choice for future HPV analysis of archival Pap-stained cervical smears. © 2000 Cancer Research Campaign


*British Journal of Cancer* (2000) 82(**8**), 1421–1426
© 2000 Cancer Research Campaign
DOI: 10.1054/ bjoc.1999.1128, available online at http://www.idealibrary.com on IDEAL®

### A simplified and reliable HPV testing of archival Papanicolaou-stained cervical smears: application to cervical smears from cancer patients starting with cytologically normal smears

**MV Jacobs[1], D Zielinski[1], CJLM Meijer[1], RP Pol[1], FJ Voorhorst[2], FA de Schipper[3], AP Runsink[4], PJF Snijders[1] and JMM Walboomers[1,†]**

[1]Department of Pathology, Section of Molecular Pathology, University Hospital Vrije Universiteit, De Boelelaan 1117, 1081 HV, Amsterdam, The Netherlands; [2]Department of Epidemiology and Biostatistics, Fac. Geneeskunde Vrije Universiteit, Van der Boechorststraat 7, 1081 BT, Amsterdam, The Netherlands; [3]Department of Obstetrics and Gynecology, Hospital Walcheren, Koudekerkseweg 88, 4382 EE, Vlissingen, The Netherlands; [4]Department of Pathology, District Laboratory Zeeland, Molenwater 47, 4331 SC Middelburg, The Netherlands

**Summary** The efficacy of four methods to recover DNA from Papanicolaou (Pap)-stained archival cervical smears for optimal detection of human papillomavirus (HPV) DNA by GP5+/bioGP6+ polymerase chain reaction (PCR) was investigated. Two of the methods were based on proteinase K treatment and two based on treatment with guanidinium thiocyanate (GTC). The quality of the DNA as measured by PCR assays amplifying different sizes of the β-globin gene appeared to be superior for the GTC-based assays. Using competitive β-globin PCR assays, one of the GTC-based, assays, provisionally named High Pure PCR Template Preparation (HPPTP) assay, yielded by far the highest quantity of amplifiable DNA. It allowed the recovery of $2.2 \times 10^5$ to $3 \times 10^5$ genome equivalents in smears containing $5 \times 10^5$ to $20 \times 10^5$ nucleated cells, indicating a mean efficiency of 26% (range of 15–44%). In contrast, the other methods revealed markedly lower efficiencies varying from 1% to 10%. The use of the HPPTP assay as a reliable processing procedure was validated by demonstrating a complete agreement in HPV detection and 93% agreement in HPV typing between 39 archival Pap-stained and paired fresh-frozen cervical smears. This method was applied to 40 archival smears from ten cervical cancer patients (selected from a group of 200 patients) which had a history of 3–6 smears with the first smear being Pap 1 or 2 taken at least 5 years before cancer was diagnosed. The average time period between the first Pap 1/2 smear that contained the same HPV type as in the corresponding carcinoma and diagnosis of cervical cancer was $12.0 \pm 2.9$ years. All subsequent smears were invariably positive for the same HPV type which was also found in the cervical cancer biopsy. In conclusion, the HPPTP assay provides a reliable and efficient means to extract DNA from Pap-stained archival cervical smears for the detection of HPV DNA by PCR and would be the method of choice for future HPV analysis of archival Pap-stained cervical smears. © 2000 Cancer Research Campaign

**Keywords:** HPV; PCR; archival Pap smears

Cervical cancer, the second most common cancer affecting women, can be prevented if precancerous lesions are detected at an early stage. Despite the success of cytomorphological examination of Papanicolaou (Pap)-stained cervical smears in reducing the incidence of cervical cancer significantly, the test has still some limitations with respect to sensitivity and specificity (Koss, 1989). To date it is well-established that persistent infection with high-risk human papillomavirus (HR-HPV) is a necessary cause for the development of cervical cancer (Ho et al, 1995; Remmink et al, 1995; Nobbenhuis et al, 1999). Hence, inclusion of HR-HPV testing by sensitive polymerase chain reaction (PCR) methodology may lead to an improvement of cervical cancer screening programmes and the management of women with cervical intra-epithelial neoplasia (CIN) lesions (Meijer et al, 1998). In this respect, knowledge about the time interval between HR-HPV infection and cervical cancer diagnosis is crucial to determine rescreening intervals for women that present with an HR-HPV-negative cytologically normal cervical scrape. This can only be achieved by accurate retrospective analyses of Pap-stained cytologically normal cervical smears from women who developed cervical cancer, since for ethical reasons prospective follow-up studies are not allowed.

The feasibility of such a retrospective analysis largely relies on the recovery of sufficiently representative DNA from archival material in order to score HPV DNA presence in a reliable manner. As a first step towards retrospective analysis of Pap smears, our group has recently compared the efficacy of several DNA extraction procedures on Pap-stained cells in reconstruction experiments (de Roda Husman et al, 1995). At that time, a method based on guanidinium thiocyanate (GTC) treatment and nucleic acids capturing on silica beads appeared to be superior and allowed for the detection of HPV DNA in false-negative archival cervical smears of women who developed cervical cancer (Walboomers et al, 1995). However, it is still questionable whether this extraction procedure is the most efficient and less labourious. Therefore, the aim of this study was to extend our previous efforts to evaluate several DNA extraction methods, comprising two proteinase K-based and two modified GTC-based procedures on archival cervical smears. Particular attention was focused on the amount of

*Received 16 September 1999*
*Revised 23 November 1999*
*Accepted 9 December 1999*

*Correspondence to:* PJF Snijders

†JMM Walboomers passed away 2 February 2000

**Table 1** Quantitative assessment of amplifiable DNA extracted from 20 archival cytological smears of group B using one of four DNA extraction methods

| Archival smear | Storage time (years) | Pap class | Amount of nucleated cells | Extraction method | Yield of amplifiable DNA (genome equivalents) | Recovery efficiency (%) |
|---|---|---|---|---|---|---|
| 1 | 13 | 3b | $5 \times 10^5$ | Prot.K/Tris | $3.2 \times 10^4$ | 6 |
| 2 | 14 | 2 | $2 \times 10^5$ | Prot.K/Salting-out | $2 \times 10^4$ | 10 |
| 3 | 14 | 3b | $2 \times 10^5$ | GTC/diatoms | $0.6 \times 10^4$ | 3 |
| 4 | 14 | 3a | $5 \times 10^5$ | HPPTP | $2.2 \times 10^5$ | 44 |
| 5 | 13 | 3a | $6.5 \times 10^5$ | Prot.K/Tris | $1.6 \times 10^4$ | 3 |
| 6 | 13 | 3a | $6.5 \times 10^5$ | Prot.K/salting-out | $3.3 \times 10^4$ | 5 |
| 7 | 14 | 3b | $10 \times 10^5$ | GTC/diatoms | $3.1 \times 10^4$ | 3 |
| 8 | 14 | 3a | $6.5 \times 10^5$ | HPPTP | $1.8 \times 10^5$ | 28 |
| 9 | 14 | 3b | $13 \times 10^5$ | Prot.K/Tris | – | – |
| 10 | 14 | 3b | $13 \times 10^5$ | Prot.K/salting-out | $2.8 \times 10^4$ | 2 |
| 11 | 14 | 3a | $13 \times 10^5$ | GTC/diatoms | $4.4 \times 10^4$ | 3 |
| 12 | 14 | 3a | $13 \times 10^5$ | HPPTP | $2.5 \times 10^5$ | 19 |
| 13 | 14 | 3a | $13 \times 10^5$ | Prot.K/Tris | – | – |
| 14 | 12 | 3b | $13 \times 10^5$ | Prot.K/salting-out | $3.6 \times 10^4$ | 3 |
| 15 | 13 | 3a | $15 \times 10^5$ | GTC/diatoms | $2.4 \times 10^4$ | 2 |
| 16 | 14 | 3b | $13 \times 10^5$ | HPPTP | $2.8 \times 10^5$ | 22 |
| 17 | 12 | 3a | $20 \times 10^5$ | Prot.K/Tris | – | – |
| 18 | 13 | 3b | $15 \times 10^5$ | Prot.K/salting-out | – | – |
| 19 | 14 | 2 | $15 \times 10^5$ | GTC/diatoms | $2.1 \times 10^4$ | 1 |
| 20 | 14 | 3a | $20 \times 10^5$ | HPPTP | $2.9 \times 10^5$ | 15 |

–, No calculation possible since target DNA OD values did not fell within a 2-log range compared to constructs. Prot.K, proteinase K

amplifiable DNA, assessed by a recently developed competitive β-globin PCR assay (Jacobs et al, 1999) that could be recovered from Pap smears. Moreover, the representative nature of DNA extracted from Pap smears was determined by a comparative HPV PCR analysis of paired fresh-frozen and Pap-stained cervical smears. One procedure based on GTC and silica columns (the High Pure PCR Template Preparation assay) revealed by far the highest yield of amplifiable DNA, with efficiencies ranging from 15 to 44%. Using this procedure comparative HPV GP5+/bioGP6+ PCR analysis on paired Pap-stained and stored fresh-frozen smears revealed on overall HPV detection agreement of 100% and an HPV typing agreement of 93%. Application of the methodology on a series of consecutive archival smears collected prior to cancer diagnosis from ten cervical cancer patients revealed the same HPV type in the smears as was detected in the corresponding cancer biopsy specimens. From these data it can be concluded that the HPPTP assay is the extraction procedure of choice for future extensive retrospective HPV analyses of archival cervical smears.

### MATERIALS AND METHODS

### Archival Pap-stained smears and paired fresh-frozen cervical scrapes

For this study, two groups of Pap-stained archival cervical smears were selected. Group A comprised 20 randomly chosen Pap-stained smears dated between 1984 and 1986 that were retrieved from the archive of the Department of Pathology, University Hospital Vrije Universiteit, Amsterdam, The Netherlands. From these smears (varying from Pap 2 to Pap 3b) the number of nucleated cells was estimated by examination of ten representative microscopic fields distributed over the whole glass slide followed by multiplication of the counted number of nucleated cells with the total number of fields. Subsequently, group A smears were subdivided into five series of smears containing approximately equal number of cells and from each series, one smear was processed by one of four DNA extraction methods. Details are shown in Table 1.

Group B comprised 39 Pap-stained smears from which paired fresh-frozen scrape samples were also available. These smears could be selected since, in the routine screening procedure for cervical cancer in our hospital, one cervical smear is taken for cytological examination (Pap smear) and an additional scrape is taken for HPV DNA testing by PCR. This allowed us to use paired samples to assess the reliability of HPV testing after DNA extraction from archival Pap-stained smears. The 39 group B smears were selected on the basis of the HPV DNA status of the corresponding fresh-frozen samples; 13 of them were HPV-negative and 26 were HPV-positive. HPV detection and typing of the fresh-frozen samples was previously performed by the general primer GP5+/bioGP6+ PCR-EIA assay (Jacobs et al, 1997), followed by confirmation by HPV E7 type-specific (TS–) PCR assays (Walboomers et al, 1999). The group of HPV-positive fresh-frozen scrapes was representative for a diversity of 13 different HR-HPV types (HPV 16, 18, 31, 33, 35, 39, 45, 51, 52, 56, 58, 59 and 66). Besides normal cytology, the Pap-stained smears also comprised abnormal cytology to select a diversity of HPV genotypes. Details about HPV typing and Pap classes are given in Table 2. The group B smears were taken between 1988 and 1994. The fresh-frozen counterparts had been stored at –70°C in 10 mM Tris–HCl (pH 7.4).

 *© 2000 Cancer Research Campaign*

**Table 2** HPV analysis of 39 fresh-frozen samples versus corresponding archival Papanicolaou smears. Bold numbers indicate cases with discordant HPV typing results

| Sample-pair | Pair fresh-frozen smear | | Archival Pap-stained smear | | |
|---|---|---|---|---|---|
| | β-PCR | HPV | Pap | β-PCR | HPV |
| 1–13 | + | – | 1 | + | – |
| 14 | + | 16 | 3a | – | ND |
| 15 | + | 16 | 3b | + | 16 |
| 16 | + | 16 | 1 | + | 16 |
| 17 | + | 16 | 3a | + | 16 |
| 18 | + | 16 | 3a | + | 16 |
| 19 | + | 16 | 3b | + | 16 |
| 20 | + | 18 | 1 | + | 18 |
| 21 | + | 31 | 1 | + | 31 |
| 22 | + | 31 | 3a | + | 31 |
| 23 | + | 33 | 4 | + | 33 |
| 24 | + | 45 | 3a | + | 45 |
| 25 | + | 45 | 3a | + | 45 |
| 26 | + | 52 | 3a | + | 52 |
| 27 | + | 59 | 4 | + | 59 |
| | Typing agreement for single HPV infections: 100% (13/13) | | | | |
| **28** | + | 16,18 | 3a | + | 16 |
| 29 | + | 16,31 | 3a | + | 16,31 |
| 30 | + | 16,33 | 3a | + | 16,33 |
| 31 | + | 16,58 | 4 | + | 16,58 |
| 32 | + | 35,59 | 3b | + | 35,59 |
| 33 | + | 39,56 | 3b | + | 39,56 |
| 34 | + | 51,56 | 3a | + | 51,56 |
| **35** | + | 52,58 | 3a | + | 52 |
| 36 | + | 18,45,51 | 3a | + | 18,45,52 |
| **37** | + | 31,33,35 | 3b | + | 31,35 |
| 38 | + | 45,52,66 | 3b | + | 45,52,66 |
| 39 | + | 31,33,35,58 | 3a | + | 31,33,35,58 |
| | Typing agreement per HPV type for multiple HPV infections: 90% (26/29) | | | | |
| | Overall agreement for HPV detection and typing: 93% (52/55) | | | | |

ND, sample was not tested for HPV since the β-globin PCR was negative.

### DNA extraction from archival Pap-stained cervical smears

To remove the coverslips, all Pap-stained smears were processed as described previously (de Roda Husman et al, 1995). During sample processing of group B smears, HPV-negative controls consisting of 50 000 Pap-stained cells from the HPV-negative Epstein–Barr virus (EBV) cell line JY were included in between each series of five smears. All the HPV-negative controls appeared to be negative in HPV PCR analysis. From each series of group A smears, one sample was subjected to DNA extraction using one of the following procedures:

1. a proteinase K/Tris–HCl method as described by Puranen et al, (1996)
2. a proteinase K/salting-out method as described by Poljak et al (1995)
3. a GTC/silica method as described by de Roda Husman et al (1995), with the exception that larger diatoms were used instead of smaller silica particles because this appeared to improve the DNA yield in reconstruction experiments after one extraction round (data not shown)
4. the High Pure PCR Template assay (provisionally named HPPTP assay), which uses GTC in combination with silica columns, according to the manufacturers' instructions (Boehringer-Mannheim).

Equivalent aliquots of the processed samples were ultimately used for PCR analysis. Aliquots of extracted DNA from all group B smears were pre-screened by the β-globin 209 base pair PCR assay and the positive samples in this assay were further analysed for the presence of 14 HR-HPV types (HPV 16, 18, 31, 33, 35, 39, 45, 51, 52, 56, 58, 59, 66 and 68) by GP5+/bioGP6+ mediated PCR-EIA as described earlier (Jacobs et al, 1997) using HPV type-specific oligoprobes in a cocktail and individually.

### Statistical analysis

The agreement between HPV detection and typing results of paired Pap-stained and fresh-frozen smears was assessed by pair-wise comparison of test results using percentage of agreement and the Kappa (κ) statistic. κ-values express the proportion of possible agreement beyond chance. A κ estimate of less than 0.4 represent poor agreement, a κ estimate between 0.4 and 0.75 is fair to good agreement and a κ estimate of more than 0.75 is excellent agreement (Fleiss, 1981). In case of multiple HPV infections, agreement rates were determined on basis of each individual HPV type present in a given sample.

### Patient selection

From the files of the District Laboratory Zeeland, Middelburg, and the Hospital Walcheren, Vlissingen. The Netherlands, 200 patients

*© 2000 Cancer Research Campaign*

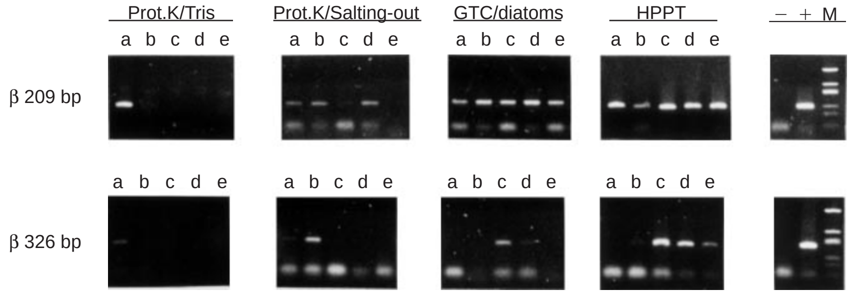

**Figure 1** PCR analysis on DNA extracts obtained with different DNA extraction methods from archival Pap-stained smears of group A using primers generating 209 and 326 base pair fragments of the β-globin gene. The Pap smears contain approximately 2–5 × $10^5$ cells (A), 6.5–10 × $10^5$ cells (B), 13 × $10^5$ cells (C), 13–15 × $10^5$ cells (D) and 15–20 × $10^5$ cells (**E**). –: β-globin PCR-negative control consisting of PCR mix only; +: β-globin PCR-positive control consisting of PCR mixture with 100 ng human placental DNA; M: size marker; pUC19 DNA digested with *Hin*f1 and *Pvu*1

were found with the diagnosis of cervical cancer. The cytological smears of these patients were retrieved from the archives and rescreened by a cytopathologist using a modified Pap classification (KOPAC). Briefly, Pap 1: normal cytology: Pap 2: inflammation; Pap 3a1: mild dyskaryosis; Pap 3a2: moderate dyskaryosis: Pap 3b: severe dyskaryosis; Pap 4: carcinoma *in situ*; Pap 5: invasive cancer. The cytopathologist did not know the patient's history. The cases selected for this study had to fulfil the following criteria: (a) the diagnosis of cervical squamous cell carcinoma is histologically confirmed, (b) at least three cytological smears starting with a Pap 1 or 2 smear had to be collected prior to the diagnosis of cervical cancer, and (c) the time period between the first Pap 1/2 smear and cervical cancer had to be at least 5 years. Only ten patients with a total of 40 smears matched these criteria. From these patients also the FIGO stage was known. The smears from these ten cases were randomly mixed with ten normal archival smears of which the corresponding fresh-frozen scrape appeared to be HPV-negative. These latter samples were used as an additional control to exclude the occurrence of contamination. The smears were subjected to DNA isolation for HPV PCR analysis. The HPV testing personnel were unaware of the cytological results of the smears. In addition, corresponding biopsies of the ten cases were cut according to a sandwich principle as described earlier (Walboomers et al, 1999) in which the outer sections were used for histological evaluation and the inner sections for HPV testing by PCR.

### RESULTS

### Qualitative and quantitative assessment of recovered DNA from archival Pap smears

For integrity analysis of extracted DNA, two PCR assays were applied using primers generating β-globin gene fragments of 209 and 326 base pairs respectively. As shown in Figure 1, the number of β-globin PCR-positive samples generally decreased with increasing amplimer length. Moreover, the integrity of recovered DNA was dependent on the method applied, with the proteinase K-based methods yielding lower numbers of samples positive in both β-globin PCR assays. Although both GTC-based methods yielded positivity for the 209 base pair β-globin PCR in all cases, amplification of a 326 base pair fragment was more efficient after HPPTP extraction (four cases versus two cases; lanes b (weak pos), c, d and e versus lanes c (weak pos) and d in Figure 1) with positive signals detectable at the gel level. These data are consistent with previous findings using reconstruction experiments of Pap-stained SiHa cells (de Roda Husman et al, 1995). Subsequently, DNA extracts were subjected to a competitive β-globin PCR-EIA generating 150 base pair fragments to determine the amount of amplifiable DNA recovered from the smears. As shown in Table 1, the HPPTP extraction assay revealed by far the highest yield of amplifiable DNA. Using this method the amount of amplifiable DNA recovered from in between 5 × $10^5$ to 20 × $10^5$ cells ranged from 2.2 × $10^5$ to 3 × $10^5$ genome equivalents. This indicates a mean efficiency of 26%, with a range from 15 to 44%. The yield of the other three methods was markedly lower. The GTC/diatoms method revealed an efficiency range from only – 1 to 3% (mean 2%).

In four of the samples processed by proteinase K-based methods the amount of extracted DNA even fell beyond the detection limit of the assay. Taken together, the yield of the remaining cases processed according to these three methods ranged from 0.6 × $10^4$ to 4.4 × $10^4$ genome equivalents, consistent with efficiencies varying from in between 1 and 10% (with a mean of 4%).

From these data we concluded that the HPPTP assay was superior in extracting DNA from Pap smears and therefore this method was chosen for further validation studies.

### Comparison of HPV detection in archival Pap smears and corresponding fresh-frozen cervical scrapes

The 39 group B smears were used to assess the representative nature of DNA extracted by the HPPTP assay. After processing according to the HPPTP assay, the Pap smears were first subjected to the β-globin 209 base pair PCR. β-globin PCR analysis revealed only one negative archival Pap smear (sample 22), resulting in a recovery efficiency of 97.4% (38/39). This sample was excluded from HPV analysis.

The remaining 38 samples were subsequently analysed by GP5+/bioGP6+ PCR-EIA after which the results were compared with those previously obtained from paired fresh-frozen scrapes. The results are shown in Table 2. Of all 13 HPV-negative fresh-frozen samples the Pap-stained counterparts were negative in the HR-HPV PCR. Furthermore, all 25 Pap smears with HR-HPV containing fresh-frozen counterparts were positive in the GP5+/bioGP6+ PCR-EIA. Thus, a complete agreement in HPV detection was obtained (κ-value: 1).

*© 2000 Cancer Research Campaign*

**Table 3** Retrospective HPV PCR analysis on Pap smears and biopsies of ten patients with cervical cancer

| Patient no. | Number of smears | | | | | | HPV type | FIGO |
|---|---|---|---|---|---|---|---|---|
| | 1 | 2 | 3 | 4 | 5 | 6 | | |
| | T/P/V | T/P/V | T/P/V | T/P/V | T/P/V | T/P/V | in biopsy | stage |
| 83 | 14.3/1/31 | 11.7/1/16 | 9.7/1/16 | 0.1/3b/16 | | | 16 | Microinvasive |
| 88 | 13.8/1/– | 10.3/1/16 | 6.2/3a2/16 | 5.0/3a1/16 | | | 16 | IIa-IIb |
| 28 | 10.8/1/16 | 8.3/3a2/16 | 7.0/2/16 | 0.0/5/16 | | | 16 | Ib |
| 26 | 9.6/1/16 | 8.4/2/16 | 6.5/3a/16 | 0.0/5/16 | | | 16 | Ib |
| 72 | 14.8/1/– | 10.8/1/18 | 9.3/3a2/18 | 8.8/2/18 | 3.5/3a2/18 | | 18 | Ib |
| 62 | 18.6/2/16 | 15.7/1/16 | 12/2/16 | | | | 16 | IIIb |
| 102 | 18.3/2/33 | 11.5/1/16 | 0.0/5/16 | | | | 16 | IIIa |
| 71 | 14.9/2/16 | 2.3/3a2/16 | 0.1/5/16 | | | | 16 | Microinvasive |
| 40 | 8.7/2/16 | 4.5/1/16 | 2.2/2/16 | 0.1/4/16 | | | 16 | Ia |
| 66 | 13.2/2/16 | 9.4/2/16 | 7.7/2/16 | 6.4/2/16 | 2.9/4/16 | 0.0/3b/16 | 16 | Ib |

T: time period in years between smear and diagnosis of cervical cancer. P: Pap class: Pap 1: normal cytology; Pap 2: inflammation, no sign of malignancy; Pap 3a1: mild dyskaryosis; Pap 3a2: moderate dyskaryosis; Pap 3b: severe dyskaryosis; Pap 4: carcinoma in situ; Pap 5: invasive cancer. V: HPV type

Further comparison of HPV typing results also showed a complete agreement in HPV typing results in case of the single HPV infections ($n$ = 13). Concerning the multiple HPV infections concordant typing results were obtained in nine of 12 sample pairs. In the three discordant cases (samples 28, 35 and 37) one of the HPV types present in the fresh-frozen scrape (HPV 18, 58 and 33 respectively) could not be detected in the corresponding Pap smear. Therefore, the HPV typing agreement rate was 93% (39/42; κ-value: 0.84). Taking all detection and typing data together, the percentage of overall agreement between fresh-frozen samples and corresponding archival Papanicolaou smear was 95% (52/55; κ-value: 0.86).

### Application of the HPPTP DNA extraction procedure on a series of smears of patients with cervical cancer

All smears showed β-globin positivity after PCR amplification. The ten control smears all appeared to be HPV-negative, in contrast to the smears of the cases, 38 of which contained HPV DNA. As shown in Table 3, in two cases (patient no. 72 and 88) a switch from HPV negativity of the first smear to HPV positivity of the second smear was observed. In two other cases (patient no. 83 and 102), a HR-HPV type (HPV 31 and HPV 33 respectively) was detected in the first smear that differed from the type present in the follow-up smears and carcinoma biopsy. In the remaining cases the same HR-HPV type could be identified in all follow-up smears and the corresponding cervical carcinoma. The average time period between the first Pap 1 or 2 smear containing an identifiable HPV type and the diagnosis of cervical cancer was 12.0 ± 2.9 years.

### DISCUSSION

The present cervical cancer screening procedure which is based on microscopic examination for the presence of abnormal cells in Pap-stained cervical smears, has not eradicated the disease in any screened population to date. One important reason for this is that the complex cervical cancer screening procedure is hampered by sampling and screening errors. This is compensated by repeating the Pap smear reading in a relative short time interval leading to too many screening rounds and overreading of slides (Richart and Barron, 1981; IARC, 1986).

Owing to the role of HR-HPV in the development of cervical cancer (zur Hausen, 1994; IARC, 1995) HR-HPV DNA testing, alone or in combination with cytology, has been advocated for novel cervical cancer screening strategies (Meijer et al, 1998). In this context, the time interval between the last cytologically normal HPV-positive cervical smear and the diagnosis of cervical cancer may provide valuable information to reconsider the rescreening interval in the current cervical cancer screening procedure. This information can be obtained from retrospective case–control studies, provided that the data is accessible. Therefore, in this study four different DNA extraction procedures were evaluated for their ability to provide sufficient PCR amplifiable DNA from Pap smears for most optimal HPV detection. Our results demonstrate that the HPPTP procedure recovers DNA of suitable quality and quantity from up to 13- to 14-year-old archival Pap smears and may form the basis for a reliable HPV detection in these specimens.

Previous studies reported a poor recovery of amplifiable DNA from Pap-stained smears using crude proteinase K-based protocols (de Roda Husman et al, 1995; Poljak et al, 1995; Walboomers et al, 1995). Also, the reproducibility of extracting amplifiable DNA was rather low (de Roda Husman, 1995). In part, this may be explained by the presence of excessive staining residues (Gall et al, 1993; Sepp et al, 1994). Still, we observed in this study that the DNA integrity hardly improved after an additional purification step (salting-out procedure) following proteinase K treatment. Since also the amount of amplifiable DNA did not markedly increase after application of the salting-out procedure, it can be concluded that these proteinase K-based procedures are not optimal for processing Pap smears. Although the integrity of extracted DNA was good in case of both GTC-based methods, the yield of amplifiable DNA recovered with the HPPTP method was markedly higher for all smears that were processed. This could be due to the fact that the HPPTP assay requires fewer manipulation steps during which DNA may be lost. Apart from a better recovery

*© 2000 Cancer Research Campaign*

the HPPTP method has the advantage that it is much less laborious and consequently also less susceptible to contamination.

Further evaluation of HPPTP-processed samples learned that the HPV detection scores were concordant with those obtained from frozen samples. Still, we observed a slight decrease in typing agreement between archival and fresh-frozen sample pairs from single (100% agreement) to multiple HPV infections (90% agreement). It is unclear whether this discrepancy reflects sampling errors or failures of the processing procedure.

The high agreement in HPV detection and typing between stained and frozen samples, including Pap 1 smears, suggests that the HPPTP-processing provides sufficient amplifiable DNA for reliable HPV detection in retrospective studies. This is particularly underlined by the fact that the mean recovery efficiency of the HPPTP assay was nearly 13 times higher than that of the GTC/diatoms method already proofed to be efficient to detect HPV DNA in false-negative archival smears of subjects who developed cervical cancer (Walboomers et al, 1995).

Application of the methodology on series of smears taken prior to the diagnosis of cervical cancer enabled in general the detection of HPV DNA of about 12 years before cervical cancer was diagnosed. In the context of HPV being a causative agent for cervical cancer, this time period fits the suggested period of 12.7 years necessary to develop invasive cervical cancer (Ootmarssen et al, 1992). The switch from HPV negativity to HPV positivity observed in two cases probably marks the time point in which infection took place while the HPV type switch in another two cases might indicate clearance of an HPV infection followed by reinfection with a different type. The observation of the continuous presence of the same HPV type throughout the series of smears from each case is very important. These results substantiate the hypothesis that persistent infection with oncogenic HPV types is a prerequisite for the development of cervical cancer (zur Hausen, 1994). However, the number of cases is small and no suitable age-matched controls have been included and therefore we are currently in the process to perform a large case–control study on archival smears. The results of this study will be presented in near future.

In conclusion, the HPPTP assay provides a reliable means to extract amplifiable DNA from fixed and Pap-stained archival cervical smears. Since this method is easy and rapid to perform, it opens the possibility to perform extensive retrospective studies to analyse the natural history of HPV infection.

### REFERENCES

de Roda Husman AM, Snijders PJF, Stel H, van den Brule AJC, Meijer CJLM and Walboomers JMM (1995) Processing of long-stored archival cervical smears for human papillomavirus detection by the polymerase chain reaction. *Br J Cancer* **72**: 412–417

Fleiss JL (1981) *Statistical Methods for Rates and Proportions*. John Wiley: New York

Gall K, Pavicic D, Pavelic J, Audy-Jurkovic S and Pavelic K (1993) PCR amplification of DNA from stained cytological smears. *J Clin Pathol* **46**: 378–379

Ho GY, Burk RD, Klein S, Kadish AS, Chang CJ, Palan P, Basu J, Tachezy R, Lewis R and Romney S (1995) Peristent genital human papillomavirus infection as a risk factor for persistent cervical dysplasia. *J Natl Cancer Inst* **87**: 1365–1371

IARC (1986) Working Group on Evaluation of Cervical Cancer Screening Programmes. Screening for squamous cervical cancer: duration of low risk after negative results of cervical cytology and its implication for screening policies. *Br Med J* **293**: 659–664

IARC (1995) Monographs on the Evaluation of Carcinogenic Risks to Humans. Human Papillomaviruses, Vol. 64, Human Papillomaviruses. International Agency for Research on Cancer: Lyon

Jacobs MV, Snijders PJF, van den Brule AJC, Helmerhorst ThJM, Meijer CJLM and Walboomers JMM (1997) A general primer GP5+/6+ mediated PCR-enzyme immunoassay method for rapid detection of 14 high-risk and 6 low-risk human papillomavirus genotypes in cervical scrapings. *J Clin Microbiol* **35**: 791–795

Jacobs MV, Walboomers JMM, van Beek J, Voorhorst FJ, Meijer CJLM, van den Brule AJC, Helmerhorst ThJM and Snijders PJF (1999) A quantitative polymerase chain reaction-enzyme immunoassay for accurate measurements of human papillomavirus type 16 DNA levels in cervical scrapings. *Br J Cancer* **81**: 114–122

Koss LG (1989) The papanicolauou test for cervical cancer detection. A triumph and a tragedy. *JAMA* **261**: 737–743

Meijer CJLM, Rozendaal L, van der Linden JC, Helmerhorst ThJM, Voorhorst FJ and Walboomers JMM (1998) Human papillomavirus typing or testing in gynaepathology. Has the time come? *Histopathology* **33**: 83–86

Nobbenhuis MAE, Walboomers JMM, Helmerhorst ThJM, Rozendaal L, Remmink AJ, Risse EKJ, van der Linden HC, Voorhorst FJ, Kenemans P and Meijer CJLM (1999) Relation of human papillomavirus status to cervical lesions and consequences for cervical cancer screening: a prospective study. *Lancet* **354**: 20–25

Ootmarssen GH van, Habbema JDF and Ballegooijen M van (1992) Predicting mortality from cervical cancer negative smear test results. *Br Med J* **305**: 449–453

Poljak M, Barlic J, Seme K, Avsic-Zupanc T and Zore A (1995) Isolation of DNA from archival Papanicolaou stained cytological smears using a simple salting-out procedure. *J Clin Pathol Mol Pathol* **48**: M55–M56

Puranen M, Saarikski S, Syrjanen K and Syrjanen S (1996) Polymerase chain reaction amplification of human papillomavirus DNA from archival Papanicolaou-stained cervical smears. *ACTA Cytol* **40**: 391–395

Remmink AJ, Walboomers JMM, Helmerhorst ThJM, Voorhorst FJ, Rozendaal L, Risse EKJ, Meijer CJLM and Kenemans P (1995) The presence of persistent high-risk HPV genotypes in dysplastic cervical lesions is associated with progressive disease: natural history up to 36 months. *Int J Cancer* **61**: 306–311

Richart RM and Barron BA (1981) Screening strategies for cervical cancer and cervical intraepithelial neoplasia. *Cancer* **25**: 543–549

Sepp R, Szabo I, Uda H and Sakamoto H (1994) Rapid techniques for DNA extraction from routinely processed archival tissue for use in PCR. *J Clin Pathol* **47**: 318–323

Walboomers JMM, de Roda Husman AM, Snijders PJF, Stel HV, Risse EKJ, Helmerhorst ThJ, Voorhorst FJ and Meijer CJLM (1995) Human papillomavirus in false-negative smears: implications for screening for cervical cancer. *J Clin Pathol* **48**: 498–503

Walboomers JMM, Snijders PJF and de Roda Husman (1996) Reply to the letter from Poljak et al. *Br J Cancer* **74**: 1509

Walboomers JMM, Jacobs MV, Manos MM, Bosch FX, Kummer JA, Shah KV, Snijders PJF, Peto J, Meijer CJLM and Muñoz N (1999) Human papillomavirus is a necessary cause of invasive cervical cancer worldwide. *J Pathol* **189**: 12–19

zur Hausen H (1994) Molecular pathogenesis of cancer of the uterine cervix and its causation by specific human papillomavirus types. In: *Human Pathogenic Papillomaviruses*, zur Hausen H (ed), pp. 131–156. Springer-Verlag: Berlin

*© 2000 Cancer Research Campaign*
